# Late mortality in survivors of childhood cancer in Hungary

**DOI:** 10.1038/s41598-020-67444-1

**Published:** 2020-07-01

**Authors:** Zsuzsanna Jakab, Miklos Garami, Katalin Bartyik, Monika Csoka, Daniel Janos Erdelyi, Peter Hauser, Attila Juhasz, Agnes Kelemen, Gergely Krivan, Peter Masat, Judit Müller, Csilla Nagy, György Peter, Imre Renyi, Istvan Szegedi, Agnes Vojcek, Marianna Zombori, Edit Bardi, Gabor Kovacs

**Affiliations:** 1 2nd Department of Pediatrics, Semmelweis University, 7–9. Tűzoltó Str., 1094 Budapest, Hungary; 2 Department of Pediatrics, University of Szeged, Szeged, Hungary; 3 Public Health Administration Service of Government Office of Capital City Budapest, Budapest, Hungary; 4 Haematology/Oncology and Pediatric Bone Marrow Transplantation Unit, Child Health Centre, Borsod-Abauj-Zemplen County Hospital, Miskolc, Hungary; 5 Pediatric Bone Marrow Transplantation Department, South-Pest Centrum Hospital, Budapest, Hungary; 6 Department of Pediatrics, Markusovszky County Hospital, Szombathely, Hungary; 7 Hemato-Oncology Unit, Heim Pal Children’s Hospital, Budapest, Hungary; 8 Department of Pediatrics, University of Debrecen, Debrecen, Hungary; 9 Department of Pediatrics, University of Pecs, Pecs, Hungary; 10 Kepler Universitätsklinikum, Linz, Austria

**Keywords:** Health care, Medical research, Oncology

## Abstract

The Hungarian Pediatric Oncology Network provides centralized treatment and population-based registration for cases of childhood cancer since 1973. We collected and analized data on late mortality, secondary malignancies and cardiac diseases in survivors (> 5 years) of childhood cancer to evaluate long-term risks. We extracted all solid tumour cases (3,650 followed up for 5–39.3 years, diagnosis: 1973–2008) from the database of the Hungarian Childhood Cancer Registry and checked against the Population Registry. Among the 301 patients who died after 5 years (8.2%) the most common causes of death were progression of primary cancer (52.5%), secondary malignancies (16%) and cardiovascular diseases (8%). Late mortality rates (SMR, total: 35,006 pyrs) showed highly elevated risk of death (SMR: 10.7 95% CI 9–12.4) for the second 5 years of follow up and moderately elevated risk for 10-year survivors (SMR: 3.5 95% CI 3–4.1). Marked differences were detected in the pattern of causes of death between diagnostic groups of primary cancer; with highest risks beyond 10 years for CNS tumours, Hodgkin disease, osteosarcoma and advanced stage neuroblastoma. The longstanding mortality risk for 5-year survivors underlines the need for tailored long-term follow-up and monitoring of late consequences according to the context of different primary diseases of childhood cancer.

## Introduction

Centralised treatment protocols and national registration for children with leukemia and solid tumours was introduced by centres of the Hungarian Pediatric Oncology Network (HPON) from 1971. Concomitantly, the Hungarian Childhood Cancer Registry (HCCR) was founded. It represents one of the oldest childhood cancer registries in Europe providing population-based registration of incidence and mortality (< 15 years). Definitive diagnosis and treatment of malignant diseases was uniformly based on consensus protocols agreed by HPON. Detailed data were collected on all cases including benign CNS (central nervous system) tumours on the basis of compulsory registration including wide range of variables including primary tumour sites, histologic and prognostic markers, details of treatment and outcome, relapses, early and late toxicities and long-term follow-up^1–3^.

Outcome of childhood cancer has shown great improvement from the nineteen sixties due to progress in multimodal therapy^4–6^. Survival rates of different diseases exceeded 50% at different time periods during the last 50 years^7,8^. As outcome of diseases showed improvement, the average survival time of patients increased. Decreasing relapse risk and improving second line therapy led to longer survival^7,8^. Consequently, the number of long-term survivors has increased corresponding to one in 870 adolescents entering adulthood in Hungary.

Why do late events happen? Either disease biology or therapeutic complications or both can be responsible. Children show high sensitivity to side effects of chemo/radiotherapy which can lead to severe complex problems, sometimes fatal events. In as few as 5–10 years an elevated risk of secondary malignancies can be detected, showing increasing trend in time with no plateau formation^9,10^. In general, one third of survivors are free of late effects, but two thirds have detectable organ damage, half of them severe and/or combined late sequlae potentially predisposing to early death^11–14^. Mortality of survivors is higher in all age groups compared to the background population in large cohort studies^9,10,12–14^. Consequently, there is a great need for long-term surveillance involving all cases.

We undertook a population-based study to collect data on adverse outcomes and late mortality in Hungarian survivors followed up by the HPON and adult practitioners after patient transfer. As Hungary is involved in the Pancare collaboration from its foundation (2009), we joined the PanCareSurfUp project funded by the European Union^15–18^. The present article provides a summary of the results on late mortality and the causes of death in Hungarian survivors of pediatric solid tumours.

## Methods

We extracted data on all solid tumour cases diagnosed between 1973 and 2008 and surviving at least 5 years (n 3,650) from the database of HCCR. In addition, we included all 5-year survivors of Hodgkin disease and osteosarcoma diagnosed below 18 years. Late mortality of patients with leukemia will be reported in a separate study. Details of childhood solid tumour diagnoses were coded according to recommendations by the ENCR (ICD-O-3: International Classification of Diseases for Oncology, 3rd edition; ICCC: International Classification of Childhood Cancer)^17,18^. CNS tumours were divided into clinical subgroups (astrocytoma, medulloblastoma/PNET (primitive neuroectodermal tumour), ependymoma, craniopharyngeoma, CNS other, CNS inoperable: without histologic examination). Neuroblastoma cases were divided into subgroups of early (I–II) and advanced (III–IV) stages. Classification of high versus standard risk patients (HR, SR) was made on the basis of the consensus protocol administered in the time period of diagnosis. Permissions were obtained from the National Ethics Committee (Semmelweis University, Budapest).

We identified patients who died on the basis of long term follow-up information routinely collected by the registry and matched them with the Population Registry (301 cases). To explore causes of death we studied detailed documentation stored on each case at HCCR. We made extensive efforts to get detailed medical documents on later events including post mortem reports. For missing cases, we contacted the registries of local councils by place of death and archives of health institutes. Death certificates (DC) were not accessible for further analysis until recently, as official statistics are produced after anonymisation in Hungary. Code of death was determined according to criteria of the International Classification of Diseases, 10th Revision (ICD-10)^18^. Specific causes of death were classified into standardised clinical subgroups: recurrence or progression of primary cancer; secondary cancers; infections; toxic events during specific anticancer therapy; neurological diseases; cardiovascular diseases; other internal diseases; external causes of death and unknown causes of death. Collection of detailed source data enabled us to take into account contributing causes of death (progression of primary disease with complications, progression of primary disease with infection). A comparison was carried out between the frequency and causes of death during 5–10 years follow-up and beyond 10 years. Probability of long-term survival was analysed using the Kaplan–Meier method (Statistica 7.0 software)^19^.

Evaluation of late mortality for all cohort members started 5–10 and > 10 years after their primary diagnosis and continued until date of death, date of censoring, or 31th December, 2015, if alive. Stata statistical program (Statistical Software: Release 13.1.StataCorp, 2013) was applied to calculate standardized mortality ratios (SMR) to quantify late mortality in the cohort. SMR was defined as the observed deaths divided by the number of expected deaths determined using country, sex, age-group and calendar period specific mortality rates. A 95% confidence interval (CI) of each SMR was calculated on the basis of Poisson probability models^20^. Absolute excess risks (AER) were determined to describe the absolute increase in risk, defined as the observed minus the expected number of deaths divided by person-years at risk, expressed as 10,000 person-years (pyrs). We calculated SMR and AER for each subgroup of childhood solid tumours, stratified by sex.

## Results

In total 3,650 5-year survivors of solid tumours (including benign CNS tumours) diagnosed between 1973 and 2008 were identified in the HCCR database (Table 1.). Median follow-up time was 16.6 years (5–39.3 years). 6% of survivors was diagnosed in the seventies, 25% in the eighties, 34.5% both in the nineties and 2000–2008. There was a slight male predominance (male/female ratio 1.25). Out of 3,650 patients surviving 5 years, 301 patients died representing an 8.2% late mortality (Table 1). Frequency of late deaths showed considerable differences according to the primary disease, exceeding 10% in Hodgkin lymphoma (HL), certain subgroups of CNS tumours (medulloblastoma/PNET, ependymoma, craniopharyngeoma, CNS other), stage III-IV. neuroblastoma, Ewing sarcoma and osteosarcoma. While late death was rare (below 5% of survivors) in Non-Hodgkin lymphoma (NHL), stage I–II. neuroblastoma, Wilms tumour, retinoblastoma and hepatic tumours.

**Table 1:** Late deaths (> 5 years) in 5-year survivors of childhood solid tumour (N = 3,650, diagnosis period: 1973–2008, Hungarian Childhood Cancer Registry), time period of death, absolute and relative distribution of causes of death in main diagnostic groups.

Survivors > 5 yrs		Death	Time period	Causes of death:									
Number: (median FUP time)		Number and ratio %	Death during 5–10 yrs FUP	PROGR DIS−/ + complications	Progr dis and infection	Infection	Toxic event Transpl-related	Cardiovascular	Secondary cancer	Neurol. disease	Other internal disease	External	Unknown
Non-Hodgkin lymphoma	367 (14.4)	11 3%	7 64%	3 27%	2 18%	1 9%	1 9%	–	1 9%	–	2 18%	–	1 9%
Hodgkin disease	473 (15.9)	49 10%	19 39%	14 (29%) 1 (2%)	4 8%	2 4%	2 (4%) 1 (2%)	10 20%	8 16%	–	–	2 4%	5 10%
Astrocytoma	529 (15.7)	41 8%	18 44%	27 66%	–	4 10%	–	–	3 7%	2 5%	1 2%	–	4 10%
Medullo-blastoma/ PNET	146 (11.4)	26 18%	22 85%	18 (69%) 1 (4%)	–	1 4%	– 1 (4%)	–	4 15%	–	–	–	1 4%
Ependymo-ma	90 (15.7)	22 24%	12 55%	16 73%	–	–	–	–	2 10%	2 10%	1 5%	–	1 5%
Craniopharyngeoma	81 (17.2)	13 16%	3 23%	2 15%	–	3 23%	–	3 23%	–	2 15%	1 7.5%	–	2 suddend 15%
CNS other types	255 (13.9)	19 7.5%	8 42%	9 47%	2 11%	–	–	2 11%	2 11%	3 16%	–	1 5%	–
Neuroblas-toma stage 1–2	202 (14.6)	2 1%	1 50%	1 50%	–	–	–	–	–	–	1 50%	–	–
Neuroblas-toma stage 3–4	179 (15.3)	23 12,8%	13 57%	15 68%	–	–	–	2 9%	4 17%	1 4%	–	–	1 4%
Wilms tumour	343 (18.7)	12 3,5%	7 58%	5 42%	1 8%	–	–	–	4 33%	–	–	2 17%	–
Softtissue sarcomas	256 (17.3)	20 7,8%	13 65%	14 70%	–	–	–	1 5%	4 20%	–	1 5%	–	–
Ewing sarcoma	108 (9.8)	20 18,5%	17 85%	14 74%	–	–	–	2 11%	3 16%	–	–	–	1 5%
Osteo- sarcoma	160 (15.2)	20 12,5%	8 40%	7 35%	–	–	1 5%	4 20%	6 30%	–	2 10%	–	–
Retinoblas-toma	127 (17.3)	4 3,1%	2 50%	–	–	–	–	–	2 50%	2* 50%	–	–	–
Primary hepatic tumours	50 (12.4)	2 4%	2 100%	2 100%	–	–	–	–	–	–	–	–	–
Carcinoma	135 (10.7)	8 6%	5 63%	5 63%	–	–	–	1 12%	2 25%	–	–	–	–
Germcell tumours	137 (16.3)	8 6%	3 38%	3 38%	1 12%	–	–	–	2 25%	–	–	2 25%	–
Other tumours	10 (10.7)	1 10%	1 100%	1 100%	–	–	–	–	–	–	–	–	–
All cases	3,650 (16.6)	301 8.2%	161 53.5%	158 52.5%	10 3%	11 4%	6 2%	25 8%	47 16%	12 4%	9 3%	7 2%	16 5%

*CNS* central nervous system, *FUP* follow-up, *Neurol* neurologic, *PROGR DIS* progression of primary disease, *PROGR DIS–/ + complications* progression of primary disease without/with complications, *Suddend* sudden death, *Transpl-related* transplantation-related, *yrs* years.

**Figure 1:**
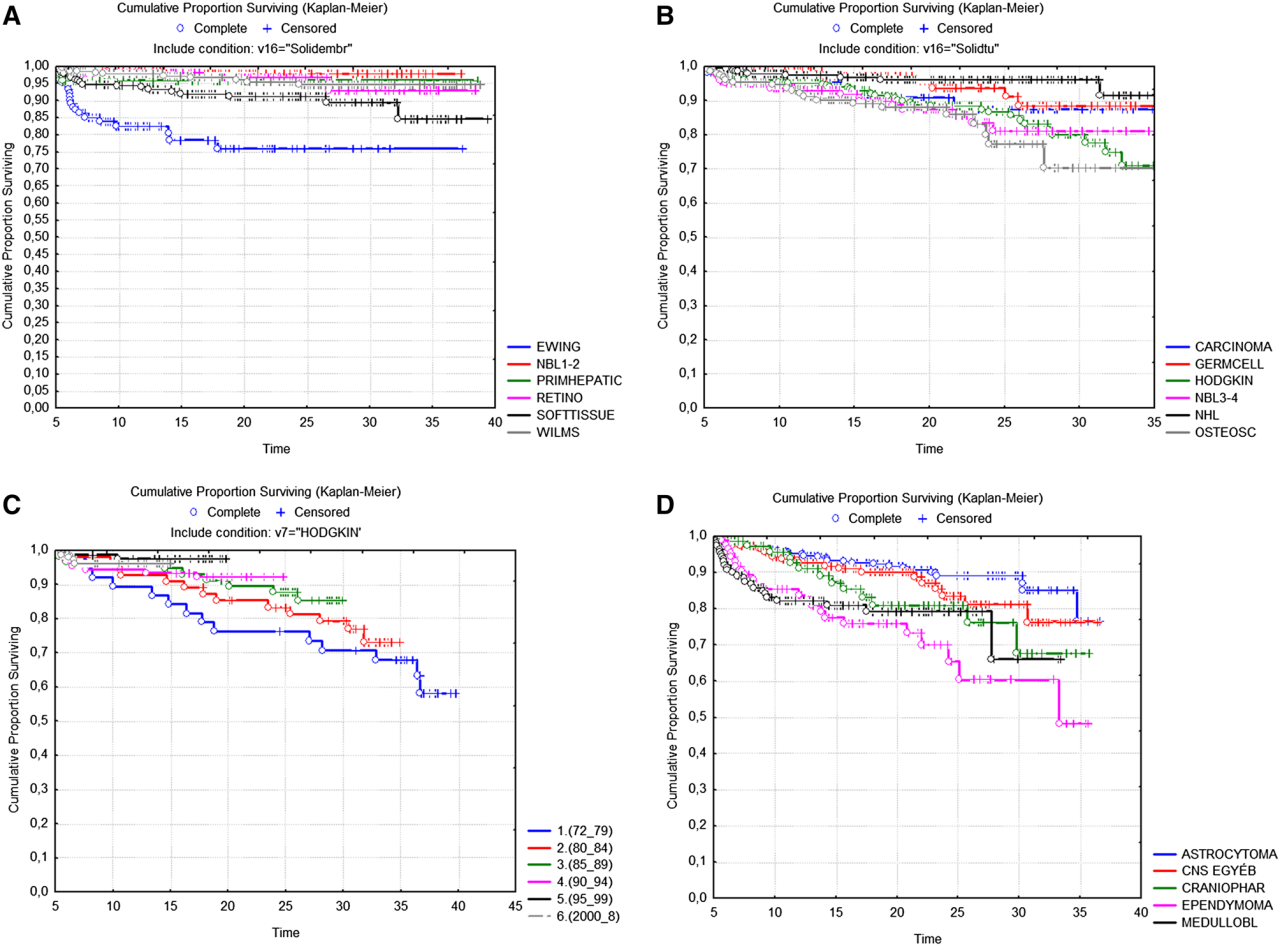
(**A**–**D**). Late overall survival (> 5 years) of 5-year survivors of childhood cancer diagnosed in 1973–2008 in Hungary, results of Kaplan–Meier analysis (Statistica 7.0, Hungarian Childhood Cancer Registry). Comparison of different subgroups: A: Ewing sarcoma, neuroblastoma stage I–II, primary hepatic tumours, retinoblastoma, soft tissue sarcoma, Wilms tumour. B: carcinoma, germ cell tumours, Hodgkin lymphoma, neuroblastoma stage III–IV, non-Hodgkin lymphoma, osteosarcoma. C: Hodgkin lymphoma, comparison of different time periods of diagnosis (1973–1979, 1980–1984, 1985–1989, 1990–1994, 1995–1999, 2000–2008). D: CNS tumours, different histologic subgroups: astrocytoma, craniopharyngeoma, ependymoma, medulloblastoma/PNET, CNS other. Abbreviations: *CNS* central nervous system, *Ewing* Ewing sarcoma, *NBL1-2* neuroblastoma stage I–II, *Primhepatic* primary hepatic tumours, *Retino* retinoblastoma, *Softtissue* soft tissue sarcoma, *Wilms* Wilms tumour, *Germ cell* germ cell tumours, *Hodgkin* Hodgkin lymphoma, *NBL3–4* neuroblastoma stage III–IV, *NHL* non-Hodgkin lymphoma, *Osteosc* osteosarcoma, *CNS egyéb* CNS other, *Craniophar* craniopharyngeoma, *Medullobl* medulloblastoma/PNET, *PNET* primitive neuroectodermal tumour.

We analysed late survival of 5-year survivors of different diagnostic groups of childhood malignant tumours according to the Kaplan–Meier method (Fig. 1A–D). Late death proved to be a very rare event in the group of survivors of NHL, Wilms tumour, retinoblastoma, stage I–II. neuroblastoma, germ cell and hepatic tumours; while in survivors of HL, Ewing tumour osteosarcoma and stage III–IV. Neuroblastoma late survival curves demonstrate a longstanding risk of dying (Fig. 1A, B). The probability of late death in osteosarcoma and advanced stage neuroblastoma beyond 20 years of diagnosis reached or exceeded 20%. When comparing different time periods, for HL survivors diagnosed in the seventies exceeded 30%, for those diagnosed in the eighties exceeded 20%, while for later groups remains below 10%, albeit with a relatively shorter observation period (Fig. 1C). The probability of late death in 5-year survivors of CNS tumours was dependent on the primary histology. For malignant subtypes (medulloblastoma/PNET, ependymoma) at 20-year follow-up exceeded 20%, even for some benign tumour types it was near to 20% (craniopharyngeoma), while for astrocytomas and the CNS-other heterogenous group it remained below 10%.

In a comparative analysis 53.5% of late deaths happened during the second 5 years of follow-up (Table 1. 4th column), which greatly exceeded the percentage of deaths observed later for most of diseases. While mortality beyond 10-year follow-up predominated in HL, astrocytomas, craniopharyngeoma, the CNS-other subgroup, osteosarcoma, and germ cell tumours.

Progression of the primary disease proved to be the most common cause of death (158, 52.5%). In a further 3% of survivors progression was complicated by infection. Secondary malignant diseases were in second place (16%), cardiovascular diseases in third (8%), followed by infections and neurologic diseases (4–4% each), other internal diseases (3%), toxic events of anticancer therapy, complications of transplantation and external causes of death (2–2% each). We could not identify the cause of late death in 5% of the patients. Distribution of these cases was random in all diagnostic groups, remaining below 5% in each. A specific time pattern of causes was observed: progression of the primary disease was predominant for the 5–10-year post-diagnosis period and to lesser extent the 10–15-year period, often coupled with infections or other complications, toxicities of anticancer therapy or transplantation-related causes. Death due to secondary malignant diseases was detected in all time periods of follow-up and most often in survivors undergoing repeated combined intensive chemotherapy, radiotherapy or both due to several relapses. Cardiac mortality was predominant in survivors of HL, bone sarcomas or craniopharyngeoma, but affected neuroblastoma cases as well, during both early and late follow-up. Mortality due to neurological diseases was most frequent in primary CNS tumours. Infections gave a considerable contribution to the mortality of survivors of lymphomas, mainly complicating progression of primary malignancy while without progression infections severely affected the craniopharyngeoma subgroup.

We performed a detailed analysis to explore correlation of late death events with the time period of diagnosis and with treatment for primary diasease and relapses. We found it indispensible as during the decades of period in study primary therapy underwent considerable changes. For diseases with high success rate of primary therapy like Wilms tumour, NHL, early stage neuroblastoma, germ cell and hepatic tumours, carcinomas, late mortality was a rare event (< 6%) with the cause of death predominantly due to progression of primary disease after one or more relapses. For diseases with limited therapy being available for relapses, like advanced stage neuroblastoma, Ewing and soft tissue sarcomas, several malignant histologic types of CNS tumours (medulloblastoma/PNET, ependymoma) late mortality was more frequent (8–25%), with death occuring during the second or third 5 year follow-up period depending on the speed of progression. In the group of survivors of HL and osteosarcoma late mortality exceeded 10% mainly due to causes not directly linked to the progression of primary disease (cardiac diseases, secondary malignant diseases). In craniopharyngeoma cardiac diseases, infections, neurologic diseases and sudden death were predominant. The most prominent correlation with time period of diagnosis seemed to be for cardiac mortality among survivors of Hodgkin lymphoma diagnosed during the seventies and eighties, and for survivors of bone sarcoma diagnosed during the eighties and nineties, in comparison to cases diagnosed later.

Late mortality rates (SMR) (Table 2) showed highly elevated near 11-fold risk of death (10.7) for the second 5 years of follow-up corresponding to an AER of 39.1 (95% CI 31.9–46.3) and moderately elevated risk for 10-year survivors (SMR:3.5, 95% CI 3–4.1; AER:16.3 95 CI 12.1–20.6). Marked differences were detected between males (SMR: 8.1) and females (SMR: 16.3) for the second 5 years of follow-up, diminishing to less then threefold in males (SMR: 2.8) and sixfold in females (SMR: 5.7) beyond 10 years after diagnosis. Comparing different decades of diagnosis the highest SMR was found for patients diagnosed in the seventies (SMR: 17.8), while for patients from subsequent decades risk corresponded to 10–11-fold elevated risk of death for the second 5 years of follow-up, falling to above 3–4 times elevated risk of death (SMR: 3.2–3.9) beyond 10 years. Correspondingly AER values fell considerably taking into account different decades of diagnosis. There was a marked difference in SMR values observed between risk groups of patients: for the high risk group 16.5-fold while for the standard risk group only sixfold risk was found in the second 5 years of follow-up, falling after 10 years to 4.6-fold and 2.7-fold, correspondingly. Similarly, late mortality rates were considerably higher for CNS tumour patients showing 18.4-fold elevated risk in comparison to 7.9-fold for non-CNS tumour cases in the second 5 years of follow-up, whereas after 10 years follow-up a 5.4-fold versus 2.8-fold elevated risk of death was observed.

**Table 2:** Standardised mortality rations (SMR) and absolute excess risks (AER) of 5-year survivors and 10-year survivors of childhood solid tumours according to sex, age at diagnosis, risk group, main diagnostic groups, with corresponding 95% confidence intervals in brackets.

	Follow-up period 5–10 yrs					Follow-up period beyond 10 yrs				
	Pyrs	Obs	Exp	SMR	AER	Pyrs	Obs	Exp	SMR	AER
All patients	35,006	151	14.17651	10.651 [9.02–12.493]	39.086 [31.89–46.282]	63,396	145	41.41742	3.501 [2.954–4.12]	16.339 [12.118–20.56]
Males	19,479	79	9.750599	8.102 [6.414–10.098]	35.551 [26.072–45.03]	35,901	86	31.12748	2.763 [2.21–3.412]	15.284 [9.376–21.193]
Females	15,527	72	4.425912	16.268 [12.729–20.487]	43.52 [32.485–54.556]	27,495	59	10.28994	5.734 [4.365–7.396]	17.716 [11.782–23.65]
**Age at diagnosis**										
< 1 yr	4,506	4	3.742565	1.069 [0.291–2.737]	0.571 [− 11.53–12.675]	8,316	15	4.973755	3.016 [1.688–4.974]	12.057 [1.523–22.59]
1–4 yrs	9,723	37	2.361386	15.669 [11.032–21.597]	35.625 [22.978–48.273]	18,803	42	8.454915	4.968 [3.58–6.715]	17.84 [10.436–25.245]
5–9 yrs	8,819	52	2.448218	21.24 [15.863–27.853]	56.188 [39.788–72.587]	16,535	39	10.6865	3.649 [2.833–5.312]	17.123 [8.768–25.479]
10–14 yrs	10,127	51	4.607548	11.069 [8.241–14.553]	45.811 [31.378–60.243]	17,197	41	15.18699	2.7 [1.937–3.662]	15.01 [6.467–23.553]
15–18 yrs	1,831	7	1.016793	6.884 [2.768–14.185]	32.677 [2.369–62.986]	2,545	8	2.115251	3.782 [1.633–7.452]	23.123 [− 1.371–47.617]
**Diagnosis period**										
1973_79	1,798	14	0.787733	17.773 [9.716–29.819]	73.483 [31.564–115.40]	6,880	28	8.773045	3.192 [2.121–4.613]	27.946 [10.671–45.222]
1980_89	8,902	47	4.760055	9.874 [7.255–13.13]	47.45 [31.61–63.29]	24,716	72	18.66532	3.857 [3.018–4.858]	21.579 [14.028–29.13]
1990_99	11,979	50	4.897524	10.209 [7.578–13.46]	37.651 [25.528–49.774]	24,345	37	11.54587	3.205 [2.256–4.417]	10.456 [4.846–16.065]
2000_08	12,327	40	3.731198	10.72 [7.659–14.598]	29.422 [18.908–39.937]	7,455	8	2.43319	3.288 [1.419–6.478]	7.467 [− 1.025–15.959]
CNS tumours	10,791	67	3.646049	18.376 [14.241–23.337]	58.71 [43.444–73.976]	18,734	62	11.46761	5.407 [4.145–6.931]	26.974 [18.006–35.941]
NonCNS tumours	24,215	83	10.53046	7.882 [6.278–9.771]	29.928 [22.1–37.755]	44,662	83	29.94981	2.771 [2.207–3.435]	11.878 [7.214–16.542]
HR	14,934	106	6.440584	16.458 [13.472–19.907]	66.666 [52.749–80.583]	26,501	80	17.29393	4.626 [3.668–5.757]	23.662 [16.367–30.957]
SR	20,072	47	7.735926	6.076 [4.464–8.079]	19.562 [12.337–26.786]	36,895	65	24.12348	2.694 [2.08–3.434]	11.079 [6.064–16.094]
NHL	3,610	6	1.465038	4.095 [1.503–8.914]	13.324 [− 1.51–28.158]	6,322	4	5.347037	0.748 [0.204–1.915]	− 2.131 [− 11.60–7.348]
Hodgkin	4,622	19	1.955754	9.715 [5.849–15.171]	10.406 [− 9.006–29.819]	8,466	32	7.103497	4.505 [3.081–6.359]	29.408 [14.93–43.885]
Astrocytoma	5,289	19	1.699856	11.177 [6.73–17.455]	32.71 [15.849–49.57]	9,725	18	6.185344	2.91 [1.725–4.599]	12.149 [2.237–22.06]
Medullobl/PNET	1,349	19	0.445332	42.665 [25.687–66.626]	137.54 [73.474–201.61]	2,070	5	1.060606	4.714 [1.531–11.002]	19.031 [− 4.279–42.341]
Ependymoma	927	13	0.337685	38.497 [20.498–65.832]	136.59 [59.377–213.81]	1,621	10	1.085432	9.213 [4.418–16.943]	54.994 [14.737–95.252]
CNS other	1,566	6	0.532012	11.278 [4.139–24.547]	34.917 [2.929–66.905]	2,334	8	1.238838	6.458 [2.788–12.724]	28.968 [3.443–54.493]
CNS (no hist)	785	5	0.328857	15.204 [4.937–35.481]	59.505 [1.868–117.14]	1,584	10	1.101806	9.076 [4.352–16.691]	56.175 [14.947–97.404]
NBLI-II	2,126	1	1.219514	0.82 [0.021–4.569]	− 1.033 [− 14.76–12.702]	3,426	1	1.73673	0.576 [0.015–3.208]	− 2.15 [− 11.61–7.314]
NBLIII-IV	1,753	10	0.97946	10.21 [4.896–18.776]	27.438 [− 9.61–64.486]	3,121	10	1.583498	6.315 [3.028–11.614]	26.967 [5.594–48.341]
Retino	1,466	2	0.649988	3.077 [0.373–11.115]	9.209 [− 12.55–30.973]	3,179	4	1.551672	2.578 [0.702–6.6]	7.702 [− 6.825–22.229]
Wilms	3,298	7	1.096119	6.386 [2.568–13.158]	14.584 [− 2.326–31.494]	7,005	5	3.668823	1.363 [0.443–3.18]	1.9 [− 6.338–10.138]
Primhepatic TU	490	1	0.279232	3.581 [0.091–19.953]	14.71 [− 30.53–59.951]	679	0	0.350947	0 [0–10.511]	− 5.169 [− 22.26–11.932]
Softtissuesc	2,283	13	1.011866	12.848 [5.427–19.451]	21.068 [− 11.06–53.205]	4,488	6	3.06223	1.959 [0.719–4.265]	6.546 [− 6.601–19.693]
Osteosarcoma	1,565	8	0.740451	10.804 [4.664–21.289]	46.387 [9.361–83.413]	2,760	11	2.127505	5.17 [2.581–9.251]	32.147 [6.417–57.877]
Ewing	952	10	0.364838	27.409 [13.144–50.407]	101.21 [34.927–167.49]	1,096	3	0.816056	3.676 [0.758–10.744]	19.926 [− 15.00–54.861]
Germ cell	1,113	2	0.438875	4.557 [0.552–16.462]	14.026 [− 13.47–41.528]	2,261	5	1.345693	3.716 [1.206–8.671]	16.162 [− 5.675–37.999]
Carcinoma	1,133	4	0.434283	9.211 [2.51–23.583]	31.471 [− 4.957–67.9]	1,619	3	1.143699	2.623 [0.541–7.666]	11.466 [− 13.17–36.109]

*AER* absolute excess risk, *CNS* central nervous system, *CNS (no hist)* central nervous system tumour with unknown histology, *Ewing* Ewing sarcoma, *exp* expected number of deaths, *Germ cell* germ cell tumours, *Hodgkin* Hodgkin lymphoma, *HR* high risk, *Medullobl* medulloblastoma, *NBLI-II* neuroblastoma stage I–II, *NBLIII-IV* neuroblastoma stage III–IV, *NHL* non-Hodgkin lymphoma, *nonCNS tumours* tumours located outside the central nervous system, *obs* observed number of deaths, *PNET* primitive neuroectodermal tumour, *Primhepatic tu* primary hepatic tumours, *pyrs* personyears, *Retino* retinoblastoma, *SMR* standardized mortality ratio, *Softtissuesc* soft tissue sarcoma, *SR* standard risk, *Wilms* Wilms tumour.

Great differences of SMR were seen between histologic groups of primary cancer with the highest risks for medulloblastoma (42.7), ependymoma (38.5) and Ewing sarcoma (27.4) for the second 5 years of follow-up, while beyond 10 years staying over fourfold risk for CNS inoperable, CNS other, ependymoma, medulloblastoma, HL, osteosarcoma and advanced stage neuroblastoma. We found no elevated risk of dying beyond 10 years follow-up in NHL, early stage neuroblastoma and primary hepatic tumours. AER values fell considerably after 10-year follow-up in several histologic groups of primary cancer but remained high (above 1.5 fold compared to the SR group) for survivors of HL, bone sarcomas, advanced-stage neuroblastoma and all CNS tumour types except for astrocytomas.

## Discussion

Childhood cancer is rare representing less than 1% of all malignant diseases diagnosed. However, it plays a major role in childhood mortality (second cause for ages 1–14 years)^4–7,21^. Age-standardised yearly incidence rates show a slowly increasing trend (average annual percent change:1.7%/year, CI 0.8–2.5%, standardised incidence:161/million pyrs, 2001–2015. HCCR, unpublished), in all major subgroups of diseases (leukemia, CNS tumours, non-CNS tumours)^4–7,21^. The data we present on long-term outcomes in chidhood cancer survivors were obtained in a population-based national study, based on 301 late deaths observed among 3,650 survivors of solid tumour, affecting 8.2% of cases surviving more than 5 years. Cure for childhood cancer is generally defined as 5 year disease-free survival after primary diagnosis. Nowadays overall cure rates exceed 70% for solid tumours in Hungary^22^. Improvement in survival rates in Hungarian patients goes in paralel with results in higly developed countries reflecting longstanding efforts undertaken by centres of HPON through the introduction of centralized consensus therapies in 1973^22^. Unfortunately, not all types of childhood cancer have such favourable prognosis, there are diseases showing very good and others with much worse response to standardized treatment^7,8,22^. The risk of relapse after 5 years is low but it can still happen in several subtypes of cancer. In cases relapsing during the first 5 years the risk of subsequent relapses is higher and the chance of cure considerably lower. Moreover, intensive combined treatment can in turn lead to increasing risk of severe early and/or late adverse effects especially in cases of repeated administration^9,10,12–14^.

Our present study, the first population-based project undertaken to evaluate late outcome in childhood cancer survivors in Hungary, highlighted the fact that childhood cancer gives considerable contribution to premature mortality. Reaching better 5-year survival rates in the seventies and eighties was coupled with a tendency of delay in deaths beyond 5 years after diagnosis. This fact is reflected in higher and decreasing mortality rates during 5–10 year follow-up, which fell during subsequent decades due to the improvement of therapeutic efficiency. To monitor changes and explore differences in frequency and causes of death we decided to split the follow-up period in two for our study: the second 5 years and beyond 10-year follow-up. Late mortality rates were highly elevated at near 11-fold risk of death compared to background mortality for the second 5 years of follow-up, dropping to 3, 5-fold risk of death beyond 10 years. Late mortality rates were the highest for those diagnosed in the first time period after the introduction of standardised treatment reducing in cohorts diagnosed later, similarly to other reports in the literature^23–27^.

Our results are similar in terms of frequency and distribution of causes of late death including late mortality rates to other population-based studies^23–27^. They confirm that the major cause of death for survivors is progression of primary disease not only in the first 5 year of follow-up but also thereafter. Owing to the availability of very detailed documentation sources, we were able to take into account contributing causes of death. Infectious complications of progressive disease and its treatment were found to considerably contribute to the risk of dying, in greatest percentage in lymphoma survivors (HL:8%, NHL: 18%).

Our study confirms that secondary malignancies and cardiovascular diseases largely contribute to early increased mortality in young adult survivors of childhood cancer^23–28^. According to our results, out of all late deaths in survivors of Hodgkin disease as many as 20% were due to cardiac diseases; and even more (28%) if cardiovascular problems were taken into account, which is higher than in other cohorts published^23–27^. This observation was most prominent among cases treated with the highest doses of radiation and cytotoxic agents between 1973 and 1989. For bone sarcoma survivors the contribution of cardiac deaths was remarkable (Ewing sarcoma: 11%, oteosarcoma: 20%), while among other diseases this was a relevant proportion only in craniopharyngeoma (23%). This is most probably in connection with the high-dose anthracycline therapy that survivors of bone sarcomas had received; while in survivors of Hodgkin disease with high-dose radiotherapy to the chest in addition to anthracycline-based chemotherapy, both given in repeated cycles. Both groups of survivors were found to run the risk of severe cardiac events including sudden death due to arrithmia/ischemia/pericarditis or severe chronic cardiac disease^23–27^. For cases followed up for craniopharyngeoma who had never been treated with cardiotoxic drugs or chest irradiation, severe obesity and/or combined hormonal abnormalities provided the main risk factors for cardiovascular morbidity. Taking into account the fact that the majority of our cohort of survivors represents the young adult age group with only a low number of survivors above the age of fourty makes the results on cardiac mortality even more alarming. On the other hand we identified certain groups of survivors which experience much lower mortality risk nearing the rate of background mortality beyond 10 years follow-up (NHL, early stage neuroblastoma, primary hepatic tumours). The great differences observed in SMR and AER in different tumour types underline the importance of optimising the long-term risk-adapted follow-up care of survivors and the indispensability of continuous collection of details on specific outcomes.

Secondary malignant diseases give a considerable contribution to the late mortality in survivors of childhood solid tumour (7–33%) in our experience. It was greatest in Wilms tumour, advanced stage neuroblastoma, medulloblastoma/PNET, Hodgkin disease, soft tissue and bone sarcomas, in subgroups treated by the highest doses of combined chemotherapy and/or irradiation. Comparing early and advanced stage neuroblastoma survivors, the frequency of late death shows great contrast. Late death was observed in only 1% of survivors of early stage neuroblastoma with no deaths due to cardiovascular disease and secondary malignancy. For advanced stages the rate of late death is nearing 13% with considerable contribution of secondary malignancies (17%) and cardiac causes (9%). These are most probably related to intensive multimodal treatment (for early stage patients combined chemotherapy was given in 50%, radiotherapy only in 8%). The main cause of death in both groups is progression of primary disease, but the greatest difference in mortality we find in the frequency and severity of late adverse effects after repetead intensive treatment^23–27^. To our knowledge this is the first longitudinal stage-specific comparison of causes of late deaths based on long-term population-based nationwide observation.

Limitations to the study include the low number of cases especially in rare diagnostic subgroups of diseases even though they include all 5-year survivors of childhood solid cancers in Hungary from 1973. Although results are similar to other recently published data, this fact makes extrapolation difficult. Moreover, due to the length of the study and considerable changes in protocols with limitations of retrospective data, it was impossible to assess the correlations of outcomes with details of the original treatment. Furthermore, it would have subdivided the diagnostic groups, thus possibly making statistical analyses impossible. Taking part in the international collaborative study PanCareSurfUp will enable us to address the above questions. The data we present represent unique documentation of the national introduction of centralised anticancer treatment in Eastern-Europe, a region generally characterised by lower survival rates in connection with lower socioeconomic indicators and suboptimal resources^2,4,6,8^. This is the first population-based longitudinal study undertaken in Eastern-Europe to report on long-term outcome of childhood cancer survivors. Due to the low number of patients in the relatively rare subgroups of germ cell tumours or carcinomas the ratio of secondary cancer deaths was unexpectedly high (25%). Apparently it was influenced by cases diagnosed with cancer predisposition syndromes. This tendency also affected retinoblastoma survivors (deaths due to secondary cancer in 50%) as bilateral retinoblastoma cases have a high risk of multiple cancers on the basis of their inherited genetic susceptibility.

The main obstacle to the study proved to be the lack of access to death certificates in the past. However, causes of death routinely documented on death certificates in Hungary are generally not very informative^29,30^. Nevertheless, as a result of longstanding collection of detailed data by the HCCR and comprehensive search performed for each missing case at all levels of the health care system failed for only a minority of cases (5%). Moreover, obtaining multiple documentation on most cases of late mortality enabled us to take into account contributing causes of death. New legislation introduced in Hungary ensures continuous access to death certificates from 2019.

We consider it a must to provide all survivors with detailed information on their primary disease and its treatment including potential risks of late side effects and the need for long-term follow-up. Transition and optimal care of survivors has to be tailored to risks determined by the primary disease, specific therapy administered and all potential organ damage^9–11,23–28,31,32^. Survivors can be divided into risk groups and suggestions can be made on their optimal individual care according to available guidelines^31^. However, scarce knowledge and experience of adult specialists on childhood cancer survivorship limit the availability of appropriate services^33,34^. Establishing optimal long-term survivor care is vital to prevent late morbidity and mortality to further improve outcome of childhood cancer.

### Ethical approval

All subjects or parents provided written informed consent authorizing the research projects connected to their treatment protocols. Ethical approval for the study was provided by the Scientific and Research Ethics Committee of the Medical Research Council (Semmelweis University, Hungary). All procedures performed in studies involving human participants were in accordance with the ethical standards of the institutional and/or national research committee and with the 1964 Helsinki declaration and its later amendments or comparable ethical standards.
